# Acute adrenal failure as the presenting feature of primary antiphospholipid syndrome in a child

**DOI:** 10.1186/1824-7288-38-49

**Published:** 2012-09-20

**Authors:** Nicola Improda, Maria Alessio, Donatella Capalbo, Giustina Russo, Ida D’Acunzo, Loredana Palamaro, Claudio Pignata, Mariacarolina Salerno

**Affiliations:** 1 Department of Pediatrics, Federico II University, Naples, Italy

**Keywords:** Adrenal insufficiency, Adrenal hemorrhage, Antiphospholipid syndrome, Thrombotic events

## Abstract

**Introduction:**

Antiphospholipid syndrome (APS) is characterized by recurrent arterial and venous thrombosis and detection of antiphospholipid antibodies (aPLs). This syndrome may be associated with connective tissue disorders, or with malignancies, but it may also appear in isolated form (primary APS). We report on a pediatric patient presenting with acute adrenal failure as the first manifestation of primary APS.

**Case report:**

A previously healthy 11-year-old boy developed fever, abdominal pain, and vomiting. An abdominal computed tomography scan showed nodular lesions in the adrenal glands. He was referred to our Department and a diagnosis of APS and acute adrenal failure was considered, based on positive aPLs (IgG and IgM), elevated ACTH levels and low cortisol levels. Other features were anemia, thrombocytopenia, elevated inflammatory parameters, hypergammaglobulinemia, prolonged partial thromboplastin time, positive antinuclear, anticardiolipin, anti-platelet antibodies, with negative double-stranded DNA antibodies. Lupus anticoagulant and Coomb’s tests were positive. MRI revealed a bilateral adrenal hemorrhage. A treatment with intravenous metylprednisolone, followed by oral prednisone and anticoagulant, was started, resulting in a progressive improvement. After 2 months he also showed hyponatremia and elevated renine levels, indicating a mineralcocorticoid deficiency, requiring fludrocortisones therapy.

**Conclusion:**

The development of acute adrenal failure from bilateral adrenal haemorrhage in the context of APS is a rare but life-threatening event that should be promptly recognized and treated. Moreover, this case emphasizes the importance of the assessment of aPLs in patients with acute adrenal failure in the context of an autoreaction.

## Background

Primary adrenal insufficiency is a life-threatening event, resulting from destruction or dysfunction of the adrenal cortex. Signs and symptoms of adrenal insufficiency appear when more than 90% of the cortex is destroyed [1]. It is a rare disease with a prevalence in developed countries of 90–140 per million, but it is thought to be even less common in childhood [2]. Signs and symptoms may be nonspecific and therefore the diagnosis is often delayed, with risk of severe morbidity or mortality [3]. Recent data suggest that the most common causes of acquired primary adrenal insufficiency are autoimmune adrenalitis and tuberculosis, whereas only 5% of the cases are related to unusual disorders [4].

Adrenal insufficiency has been described in APS and is thought to be the result of hemorrhage of adrenal glands. APS is an autoimmune disorder characterized by the persistent detection of antiphospholipid antibodies (aPLs) and various clinical manifestations, the most common being venous and arterial thrombotic events, recurrent fetal loss, thrombocytopenia, livedo reticularis and neurological manifestations. APS may occur in the context of another autoimmune disease (secondary APS), or may be a primary APS. In rare circumstances, APS is defined as catastrophic due to an accelerated form of multiorgan failure for an uncontrolled thrombosis [5].

Addison’s disease is reported in 0.4% of patients with ascertained APS [6]. On the contrary, APS is diagnosed in less than 0.5% of all patients with Addison’s disease [7]. To date, there are only a few reports in the literature of association between primary adrenal insufficiency and primary APS in pediatric patients.

Here we report on a 11-year-old boy with acute adrenal failure, due to bilateral adrenal hemorrhage, as the presenting feature of a primary APS.

### Case report

A previously healthy 11-year-old boy developed persistent fever, abdominal pain and vomiting. An abdominal ultrasound echography was normal. An abdominal computed tomography scan showed nodular lesions in the adrenal glands. He was referred to our Department, where the diagnosis of acute adrenal failure was made on the basis of the clinical phenotype, mild hyponatremia (132 mmol/l), high plasma ACTH level (961 pg/ml n.v. 10–130), low plasma cortisol levels (31,5 ng/ml n.v. 50–200), and normal aldosterone and renine levels. MRI revealed a bilateral adrenal hemorrhage. Routine analysis revealed anemia, thrombocytopenia, elevated inflammatory parameters, hypergammaglobulinemia and prolonged partial thromboplastin time. A subclinical hypothyroidism (SH) was diagnosed on the basis of mild increase of TSH with normal levels of FT4 [8,9], in the absence of thyroid autoantibodies. Subsequent diagnostic work-up showed positive antinuclear, antiphospholipid (IgG 20,9 IgM 27,3 n.v. <10), anticardiolipin antibodies (56,6 U/ml n.v. 0–20), anti-platelet autoantibodies and negative double-stranded DNA antibodies. Lupus anticoagulant and Coomb’s test were positive. A diagnosis of antiphospholipid syndrome was performed.

Main causes of primary adrenal insufficiency were ruled out. In particular, autoimmune Addison, a frequent cause of acquired adrenal insufficiency also in the context of APECED or others autoimmune syndromes [10-13], was ruled out by clinical and biochemical evaluation [14]. Family history was negative for APS or any other autoimmune diseases.

A treatment with intravenous metylprednisolone, followed by oral prednisone and anticoagulant, was promptly started, resulting in a progressive improvement. TSH levels spontaneously improved not requiring L-Thyroxine therapy [15,16]. Two months later, mineralcorticoid deficiency was diagnosed on the basis of hyponatremia, low aldosterone and elevated renine levels (>300 pg/ml), requiring fludrocortisone replacement therapy. The patient is currently on cyclosporine therapy because of the recurrence of thrombocytopenia and positivity of lupus anticoagulant and Coomb’s tests, and antiphospholipid antibodies. His general conditions are stable.

## Discussion

Adrenal failure is a rare but potentially life-threatening event. In childhood it generally presents with nonspecific signs and symptoms, such as fatigue, malaise, abdominal pain, nausea and vomiting. Therefore, diagnosis and treatment may be often delayed [3].

Main causes of congenital and acquired primary adrenal insufficiency are reported in Table 1[17]. Adrenal hemorrhage is a rare but well-known cause of adrenal insufficiency. In a recent retrospective study on the presentation of primary adrenal insufficiency in childhood, adrenal haemorrhage was reported in only 2 of 77 cases (3%) detected between 1999–2010 [3]. Adrenal glands are highly susceptible to hemorrhagic damages [18].

**Table 1:** Main causes of primary adrenal failure

	
Congenital	CAH
	Congenital adrenal hypoplasia
	ACTH resistance
	Glucocorticoid resistance
	Metabolic diseases (Adrenoleukodystrophy, Zellweger, Smith-Lemli-Opitz, Wolman disease)
Acquired	Autoimmune adrenalitis
	- Isolated
	- Autoimmune polyendocrinopathy
	- syndrome type 1
	- Autoimmune polyendocrinopathy syndrome type 2
	Hemorrhage/infarction
	- Trauma
	- Waterhouse-Frederickson syndrome
	- Anticoagulation
	- Thrombosis (APS, Thrombophilia)
	Drug effects (Aminoglutethimide, mitotane, ketoconazole, medroxyprogesterone)
	Infection
	- Viral: HIV, cytomegalovirus
	- Fungal: coccidiomycosis, histoplasmosis, blastomycosis, cryptococcosis
	- Mycobacterial: tuberculosis
	- Amebic
	Infiltrative (Hemochromatosis, histiocytosis, sarcoidosis, amyloidosis, neoplasm)

The presence of antiphospholipid antibodies is a major risk factor for hemorrhagic infarction of the adrenal glands as a consequence of a thrombotic event [19]. The detection of antiphospholipid antibodies in a subject with thrombosis should lead to suspect a diagnosis of APS. Criteria to confirm the diagnosis of APS include at least one clinical sign, such as vascular thrombosis or pregnancy complications, and one biochemical criterion such as anticardiolipin IgG or IgM antibodies, lupus anticoagulant of IgG or IgM classes detected in two occasions at least 6–12 weeks a part [7,20]. Recent evidence demonstrates that aPLs may recognize the β2-glycoprotein I bound to the surface of resting endothelial cells, whose activation switches them to a procoagulant and pro-adhesive phenotype. Subsequently, the great majority of clinical signs of APS, including adrenal failure, are related to recurrent venous, arterial or small-vessel thrombosis [21,22].

Besides vascular occlusion a great variety of nonthrombotic manifestations may be associated [5,20]. Our patient had hematological complications, such as hemolytic anemia and thrombocytopenia. The clinical spectrum of antiphospholipid syndrome is very broad and primary adrenal failure is considered a rare manifestation, particularly, in childhood.

Espinosa and colleagues reviewed all cases of primary adrenal insufficiency associated with APS described in the literature from 1983 (when APS was first defined) through March 2002. Of the 86 patients identified, 6 (3 males) were less than 18 years old; 5 of these cases had a primary APS and only one patient was diagnosed with a SLE-like syndrome. Moreover, in two patients Addison’s syndrome was the first manifestation of APS, while in two children adrenal insufficiency developed in the context of catastrophic APS. Furthermore, in two cases precipitating factors were identified, such as neonatal infection and pneumonia [1].

In a recent study, Presotto et al. identified, throughout a computer-assisted search of the literature from 1988 through January 2005, 19 cases of primary adrenal insufficiency as the heralding symptom of primary APS. 4 patients (2 males) were less than 18 years old and in 1 case a precipitating factor was identified [7]. Massive bilateral adrenal hemorrhage has been also described in a newborn with primary antiphospholipid syndrome hypothesized basing on the persistent detection of anticardiolipin antibodies after 6 months of life [18].

In our case, bilateral adrenal haemorrhage appeared to be the first expression of a hypercoagulable state due to the APS in the absence of any precipitating factor.

Of note, in our patient, the fact that the mineralcorticoid activity was damaged only later suggested a centrifugal progression of adrenal impairment from the zona reticularis to the zona glomerulosa, consistent with a thrombosis of the central adrenal vein(s).

## Conclusion

In conclusion, the development of acute adrenal failure due to bilateral adrenal haemorrhage in the context of antiphospholipid syndrome is a rare but life-threatening event that should be promptly recognized and treated. Moreover, our case emphasizes the importance in the assessment of antiphospholipid antibodies in all patients with rapidly progressive acute adrenal failure in particular when other autoimmune signs and symptoms are present.

### Consent

Written informed consent was obtained from the parents of the patient for publication of this Case report and any accompanying images. A copy of the written consent is available for review by the Editor-in-Chief of this journal.

## Abbreviations

APS: Antiphospholipid syndrome; aPLs: Antiphospholipid antibodies.

## Competing interests

The author(s) declare that they have no competing interests.

## Authors’ contribution

All authors have equally participated in drafting of the manuscript and/or critical revision of the manuscript for important intellectual content. All authors read and approved the final manuscript.
